# Interfacial Failure in Stitched Foam Sandwich Composites

**DOI:** 10.3390/ma14092275

**Published:** 2021-04-28

**Authors:** Yue Hu, Jun Zhu, Jihui Wang, Yibo Wu

**Affiliations:** 1 School of Materials Science and Engineering, Wuhan University of Technology, Wuhan 430063, China; aa76853840@163.com; 2 Luoyang Ship Material Research Institute, China State Shipbuilding Corporation Limited, Luoyang 471023, China; zhuj1109@163.com (J.Z.); wuyibo@725.com.cn (Y.W.)

**Keywords:** foam sandwich composites, reinforcement stitching, critical strain energy, vacuum-assisted molding, fracture process

## Abstract

In this paper, the use of a customized automatic reinforcement stitching equipment was demonstrated. The stitching of foam sandwich composite preforms was achieved to obtain structures with improved interfacial properties. The effect of different stitching spacings on the crack propagation process in glass fiber reinforced plastics (GFRP)/foam sandwich composite interfaces was examined by Mode-I Cracked Sandwich Beam (CSB) fracture tests. The load–displacement curve, the crack propagation process, and the release rate of critical strain energy were analyzed. The CSB fracture test results show that the stitching treatment with different stitching spacings increase the peak load and fracture displacement. Furthermore, it was found that the mechanism of crack propagation is changed by the stitching process. The release rates of the critical strain energy in specimens with 0- and 10-mm stitch spacings were evenly distributed, with an average of 0.961 kJ/m^2^ and 1.667 kJ/m^2^, respectively, while the release rates of critical strain energy in specimens with 6-mm and 8-mm stitch spacings were linearly distributed. The CSB fracture tests confirmed that the best suture spacing was 8 mm. Based on these results, the mechanism of crack propagation and the toughening mechanism of the resin column could be revealed.

## 1. Introduction

With the development of structural optimization and low-cost manufacturing technologies, foam sandwich structures have been increasingly used in aerospace, marine, automotive, and other civilian fields [1,2,3,4]. Compared with traditional concrete, steel and other building materials, foam sandwich composites have significant advantages such as good sound and thermal insulations, high corrosion resistance, and low cost [5,6]. Traditional foam sandwich composites commonly exhibit a panel structure of high flatness with low water absorption properties, as compared to honeycomb sandwich panels. However, compared with honeycomb sandwich structures, foam sandwich structures have lower fracture resistance, which limits the application potential of such sandwich structure core materials [7,8].

The problem of panel and core material peeling caused by the common interface crack propagation of sandwich structures has attracted widespread global research attention. However, studies on the fracture toughness of sandwich structures are limited. Pan et al. [9,10] conducted a theoretical derivation and performed equivalent mechanical model calculations for large-opening stitching hole composite laminates, a unit cell model was established for stitch damage of the stitching reinforcement for composite laminates containing a large circular hole. Li et al. [11] obtained the engineering elastic constants of the sutured laminates by vibration tests, where the results indicated that the sutured stitches caused a significant deterioration of the engineering constants of the material. Wang et al. [12] extended the use of the technique of short-cut fiber toughening of composites laminate interlaminates to interfaces of sandwich structures. It was found that the damage tolerance and residual load bearing strength of the interfaces of short-cut fiber toughened specimens were significantly increased compared with those of untoughened specimens. Yudhanto et al. [13,14] investigated the damage characteristics of 3D stitched composites in in-plane tension and compression with different stitching parameters by tensile and compression tests, where the results showed that the stitching treatment could effectively increase the tensile strength of composites and reduce the compressive strength of laminates. This could be related to the suppression of edge delamination and in-plane fiber bending. Zheng et al. [15] investigated the effect of full thickness stitching on the mechanical response of foam sandwich structures and found that the in-plane and out-plane properties of the structures were significantly improved after full thickness stitching. Ji et al. [16] combined diagonal seam and Z-pin reinforcement, where the effects of different diagonal seam and Z-pin ratios on the compressive properties, tensile properties and shear properties were studied. Ma et al. [17] developed an equivalent mechanical model of the resin column by finite element simulations and verified the correctness of their multi-scale analysis by low-velocity impact tests. Francesconi et al. [18] conducted a low-velocity impact test on stitched laminates using the finite element method and investigated the in-plane and interlaminar damage processes and the inhibitory effect of sutures on delamination. Tan et al. [19] investigated the effect of suture density on the compression properties of laminates by the finite element method, where the results showed that a certain suture density could effectively reduce the impact delamination area and thus improve the post-impact compression strength. Glaessgen et al. [20] performed an analysis of the fracture mechanics based on the strain energy release rates to investigate the effect of stitching in debonded sandwich beam configurations. From their results it could be concluded that increasing stitch stiffness, stitch density and debond length decrease the strain energy release rates for a fixed applied load. Funari et al. [21] used a nonlinear model to investigate the performance of composite sandwich structures with transversely compressible core, under static and dynamic loading conditions. In addition, the effects of loading rate and sandwich characteristics on both static and dynamic frames were parameterized. Feldfogel et al. [22] studied the debonding process of tiles by combining experimental and numerical methods, and compared the experimental and numerical results. This comparison showed that the numerical model can closely track the debonding evolution and capture various phenomena observed in the experiments.

Currently, there are few studies on suture and interface enhancement techniques available, since most of the studies on suture methods are focused on full-thickness. For the application of foam sandwich composites in engineering, there are some products with large thickness, where it is, thus, difficult to suture them to the full thickness. On the other hand, chain stitching is more suitable for large curvature components and all kinds of special-shaped structural materials, where the bottom line does not need to be replaced frequently. To achieve interface reinforcement, only part of the resin column with sutures near the interface can provide a significant interface toughening effect. The disadvantages of chain sutures are in fact quite obvious. In the process of conventional suturing, the suture is flexed for many times, which leads to low suture efficiency. However, the custom-built automatic opening and reinforcing sewing equipment developed for the study presented solves this problem and greatly improves the efficiency of suturing [23]. Compared with the traditional full-thickness sutures, the partial thickness suture treatment proposed in this paper can not only solve the interface problem of sandwich structures, but also reduce the weight added. Furthermore, our suture technique proposed can be more suitable for application to large-thickness and large-curvature structural parts and, thus, it is useful for a wider engineering application range. Furthermore, the mechanism of the novel partial thickness suture process and the corresponding crack propagation and the toughening mechanism of the suture were investigated in detail. Mode-I Cracked Sandwich Beam (CSB) fracture tests were carried out to study the failure mechanism. Preforms of glass fiber reinforced plastics (GFRP)/foam sandwich composites with toothed chain stitching were prepared using a customized SUPER TUFTING 5000 (Luoyang Ship Material Research Institute, Luoyang, China) automatic opening and reinforcement stitching equipment. The Mode-I CSB fracture test specimens were fabricated by vacuum-assisted molding. Fracture tests were conducted using a CSB test configuration. The article not only presents a novel suture method, but also contains the detailed test results of the obtained samples. The mechanical performance, crack propagation mechanism and release rate of critical strain energy of sandwich structures were investigated by combining experimental results with scanning electron microscope (SEM) (Field Electron and Ion Company, Hillsboro, OR, USA) images for different stitch spacings. The effects of stitching on crack propagation were established, which can provide a basis for future studies concerning the design of foam sandwich composites with different parameters.

The work presented is structured into the following four sections: (1) The research status of stitched sandwich composites and their interfacial properties are introduced. (2) A design of a new type of preparation method for stitched sandwich structure is described in detail. Furthermore, the preparation process of specimens for CSB fracture tests is described and the results of such CSB fracture tests are presented. (3) The test results are analyzed from three aspects: (i) load–displacement curves, (ii) crack propagation process and (iii) strain energy release rate. (4) A summary and a discussion of the research findings presented in this work.

## 2. Experimental Program

### 2.1. Raw Materials

Biaxially oriented alkali-free E glass fiber cloth with a surface density of 400 g/m^2^ and ≈0.35 mm thickness was obtained from Sinoma Technology Co., Ltd. (Nanjing, China). The stitching thread used was a 2 × 1000 D twisted aramid yarn with a thread density 1100 Tex of 1.1 g/m from Changzhou Gaoyuan Chemical Co., Ltd. (Changzhou, China). The core material was a P100 polyvinyl chloride (PVC) foam with a density of 100 kg/m^3^, a thickness of 30 mm, purchased from Changzhou Tiansheng New Material Co., Ltd. (Changzhou, China). The resin was developed in-house containing a low viscosity vinyl ester resin provided by Luoyang Ship Material Research Institute, China State Shipbuilding Corporation Ltd. Poly tetra fluoroethylene (PTEF) film was provided by Shanghai Daoguan Rubber & Plastic Hardware Co., Ltd. (Shanghai, China). The equipment and materials used for vacuum assisted resin infusion (VARI) included release cloths, guide network, spiral tubes, resin tubes, vacuum bags, unidirectional permeable membrane, sealing tapes, vacuum pumps, and glass molds.

### 2.2. Preparation of GFRP/Foam Sandwich Composites Suture Preforms

**Figure 1:**
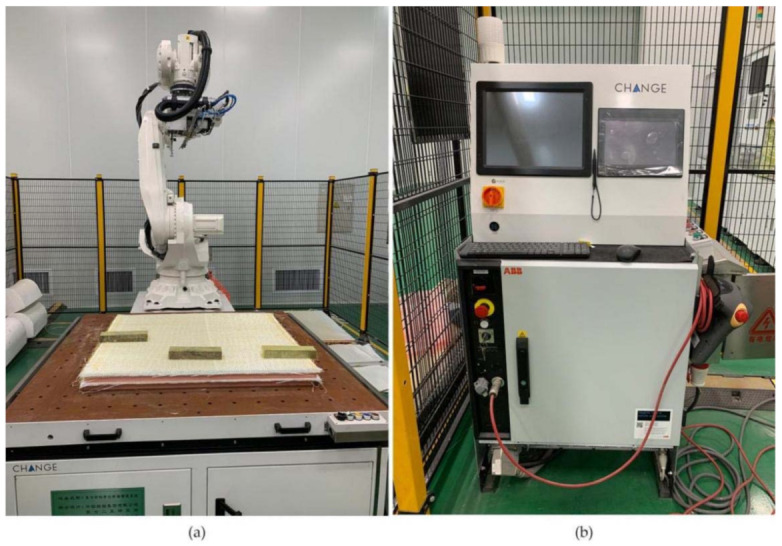
Suture equipment. (**a**) Opening and reinforcing seam laying equipment. (**b**) Console.

**Figure 2:**
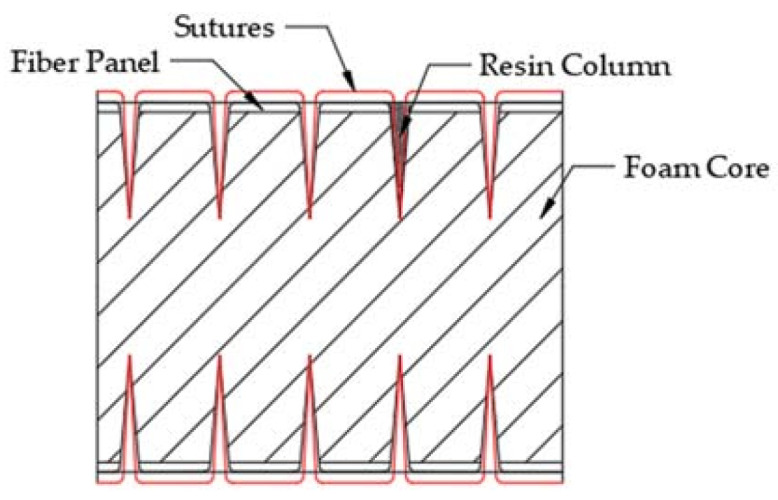
Schematic of the GFRP/foam sandwich composite suture profile structure.

A self-customized SUPER TUFTING 5000 automatic opening and reinforcing seam laying equipment (Luoyang Ship Material Research Institute, Luoyang, China) was used to prepare GFRP/foam sandwich composite suture preforms. The resin flowed into the suture space during this perfusion process and eventually solidified to form resin columns. Figure 1 shows the suturing equipment and console. Nine layers of biaxially oriented alkali-free E glass fabric were placed on the foam core, where the fiber layer of the upper and lower panels can be expressed as [90/0]_9_. The suture parameters were programmed into the controller console in terms of the stitch spacing and suture depth. Figure 2 shows a schematic drawing of the GFRP/foam sandwich composite suture profile structure.

### 2.3. VARI Processing of GFRP/Foam Sandwich Composites

**Figure 3:**
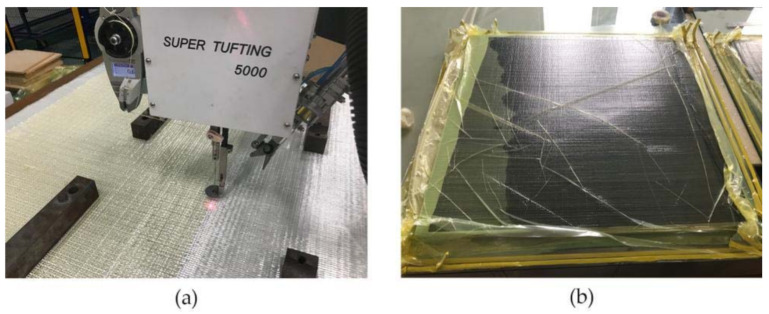
Preparation of GFRP/foam sandwich composites. (**a**) The stitching process. (**b**) Vacuum assisted resin infusion.

After the GFRP/foam sandwich composite preforms were stitched, the specimens were treated using the VARI process, as follows:

(1) Release wax was applied to the tempered glass and the preform was placed on the tempered glass plate. (2) The guide network was paved, the cloth released, and the inlet and outlet drains were operated. (3) The specimens were sealed in a vacuum bag and sealing tape. (4) The vacuum pump was turned on and vacuum injection was started. (5) After the curing process was completed, the mold was released and the specimen cut. Figure 3 illustrates the suturing and vacuum-assisted forming processes.

In the VARI process, the adhesive effect of the resin resulted in the panel fibers of the unsutured specimens being bonded to the foam core, which finally led to the formation of wholes after the curing process. In the sutured specimens, under the influence of negative vacuum pressure, the resin would flowed readily into the suture gap to form a resin column with the suture after curing.

**Figure 4:**
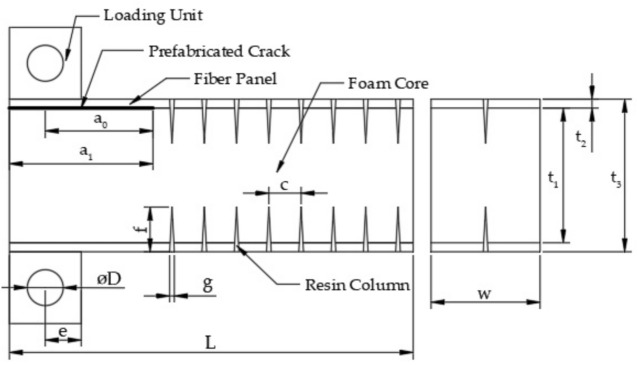
GFRP/foam sandwich composite specimens with prefabricated cracks for CSB testing.

During the specimen preparation process, cracks were prefabricated using a 60 mm wide PTEF film to separate the interface layers. Figure 4 shows the GFRP/foam sandwich composite CSB specimens with prefabricated cracks. The diameter of the suture needle was 1.85 mm. All other stitching parameters are illustrated in Figure 4; with w = 25 mm, t_1_ = 30 mm, t_2_ = 3.4 mm, t_3_ = 36.8 mm, a_1_ = 60 mm, a_0_ = 50 mm, D = 10 mm, e = 10 mm, L = 180 mm, f = 10 mm, and g = 1.85 mm. The stitch spacing (c) was set to 6 mm, 8 mm and 10 mm.

### 2.4. CSB Fracture Testing of GFRP/Foam Sandwich Composites

Four groups of specimens were fabricated. The unsutured test group was FH-0, and the suture groups included FH-6, FH-8 and FH-10, corresponding to specimens with suture spacings of 6, 8, and 10 mm, respectively, with 3 nominally identical specimens in each group. These three specimens were set up in each group to investigate reproducibility. The labels of FH-X-Y represent specimen with a suture spacing of X mm, whereas Y = 1, 2, 3 correspond to the 3 specimens fabricated in each group. The specimen size was 180 mm × 36.8 mm × 25 mm.

**Figure 5:**
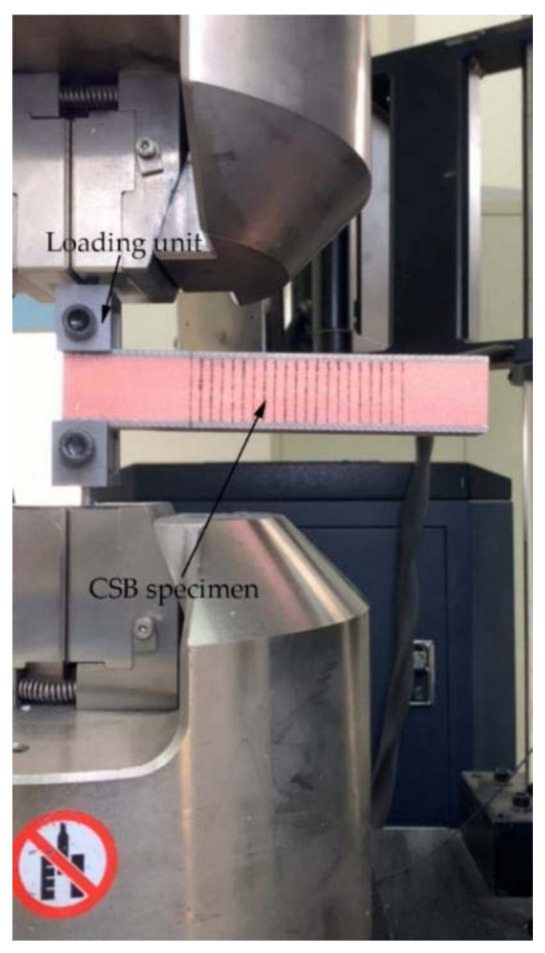
Setup of CSB testing of GFRP/foam sandwich composite specimens.

For CSB fracture testing, the surfaces of the CSB loading unit was cleaned to prevent the loaded specimen from detaching. For this purpose, the CSB testing surfaces were polished with sandpaper and cleaned with acetone to remove dirt. AB glue was applied to attach the CSB test specimens to the loading unit surfaces. During this process, the thickness of the glue layer was kept uniform and the center of the loading unit was aligned with the center of the specimen panel, followed by drying in the oven. The oven temperature was set to 35 degrees Celsius, and the specimens were continuously dried for 2 h to fully solidify the glue. Finally, the auxiliary fixture was assembled onto the fixture of the INSTRON-5567 electronic universal material testing machine (Instron Corporation, Norwood, MA, USA). Figure 5 shows the assembly of the GFRP/foam sandwich composite specimens for CSB testing.

The test method is conducted in accordance with ASTM D5528-2013 [24], where the GFRP/foam sandwich composite specimens with PTEF film prefabricated cracks were clamped to the loading unit as shown in Figure 5. The CSB test specimens were loaded at a rate of 1.3 mm/min. The loading caused the specimens to open from the prefabricated cracks, and the crack propagation process was captured using HD video equipment, where the delamination length and load–displacement curve were recorded. The release rate of critical strain energy was calculated using the modified beam theory equation [24].

## 3. Results and Discussion

### 3.1. Load–Displacement Curves

**Figure 6:**
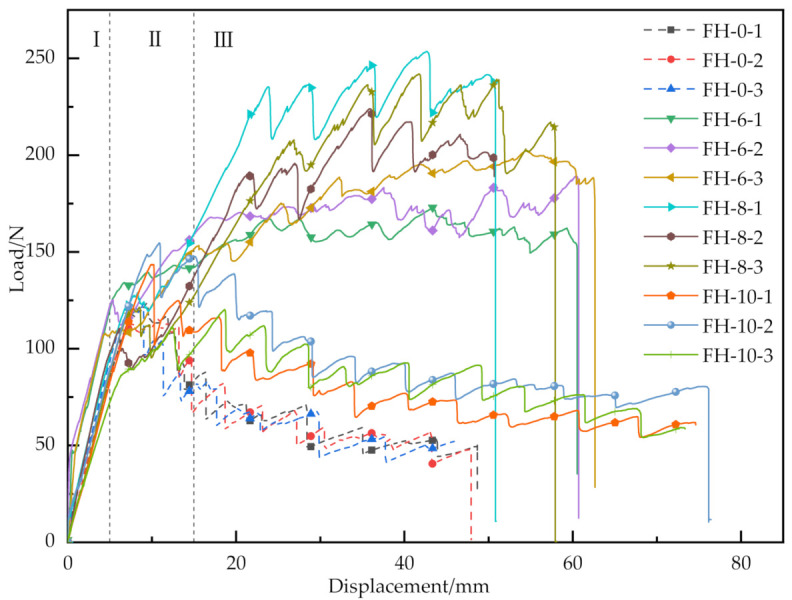
Load–displacement response of GFRP/foam sandwich composite specimens using CSB testing.

**Figure 7:**
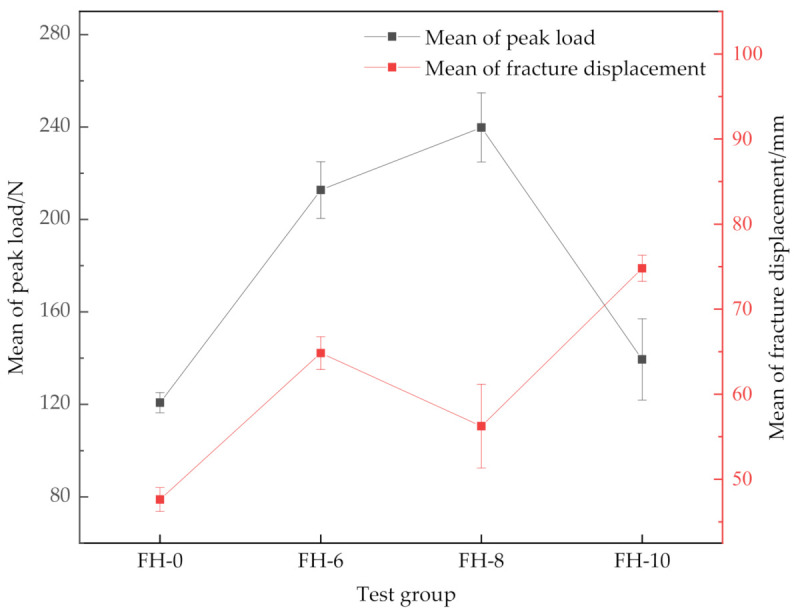
Average peak load, average fracture displacement and standard deviation in GFRP/foam sandwich composites using CSB testing.

The CSB on process and the release rate of critical strain energy of the material with different suture parameters were investigated by evaluating the testing videos, load–displacement curves and scanning electron microscope (SEM) images. Figure 6 shows the load–displacement curves of all GFRP/foam sandwich composites. Figure 7 gives the average peak load, average fracture displacement and standard deviation of GFRP/foam sandwich composites.

The specimen loading process could be divided into three stages: linear increase of the load (I), crack initiation (II), and crack propagation (III) [25]: in the linear increasing stage (I), the load–displacement curves of all specimens were approximately linear before the initial crack initiation, when the prefabricated cracks started opening. In the crack initiation stage (II), the crack initiation occurred at the prefabricated crack tip and the load–displacement curve started to increase non-linearly. In the crack propagation stage (III), local instability propagation was detected. This was manifested by the crack suddenly propagating when the increasing load reached a certain critical value. At this point the load dropped sharply, and, as the test continued, the load would gradually rise again. At this point the crack suddenly expanded, and the load dropped sharply again. This cycle was repeated several times. Finally, the crack propagated to the end of the specimen, the panel was separated from the core, and the test ended.

As shown in Figure 6 and Figure 7, the stitching treatment improved the peak load and average fracture displacement of GFRP/foam sandwich composite, as demonstrated by the CSB testing. The average peak loads for the FH-6, FH-8 and FH-10 specimen groups were 188.21, 239.81 and 139.47 N, respectively. Compared with the average peak load of 120.75 N for FH-0, the respective increases correspond to 55.9%, 98.6% and 15.5%, respectively. The average fracture displacements for the FH-6, FH-8 and FH-10 specimen groups were 61.34, 56.25 and 74.80 mm, respectively. Compared with the average fracture displacement of 47.62 mm for FH-0, the respective increases correspond to 28.8%, 18.1% and 57.1%, respectively. It is interesting to note that significant differences in the CSB test peak loads and fracture displacements were detected between the 6 mm spacing (FH-6) and 8 mm spacing (FH-8) suture treatments.

**Figure 8:**
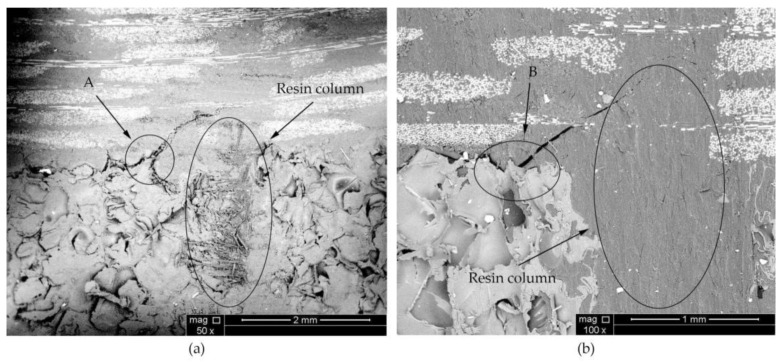
SEM images of (**a**) FH-6, (**b**) FH-8 specimen with cracks deflected by the resin column (A and B are the deflection points).

Considering the energy balance, the process of local instability propagation of the cracks (III) can be identified to occur in two stages: potential energy accumulation and potential energy release. When the potential energy accumulation of the material reaches a critical value, the potential energy begins to be released and the crack starts to propagate unsteadily. From an experimental point of view, the suture density of FH-6 is larger, so the accumulated potential energy is higher and the crack should extend farther. In practice, however, the accumulated potential energy is not completely released before the next cycle starts. This can be attributed to the fact that the suture density of FH-6 is larger and the crack propagation process is hindered by the next resin column to be extracted earlier. Although FH-8 also shows this effect, the frequency is lower. In the SEM images in Figure 8, the crack deflects at A and B due to the influence of the resin column.

According to Figure 6, the area enclosed in the linear increase phase (I) of the load curve was significantly longer for FH-6 than for FH-8, which indicated that the intensive suturing would increase the initial fracture potential energy of the material. In the crack propagation phase (III), the crack propagation process in FH-6 was prematurely terminated due to the earlier and higher frequency of the resin column to be pulled out, which is consistent with the load–displacement curves, i.e., for FH-6 the curves show more peaks and shorter spans, whereas for FH-8 they display fewer peaks and larger spans.

**Figure 9:**
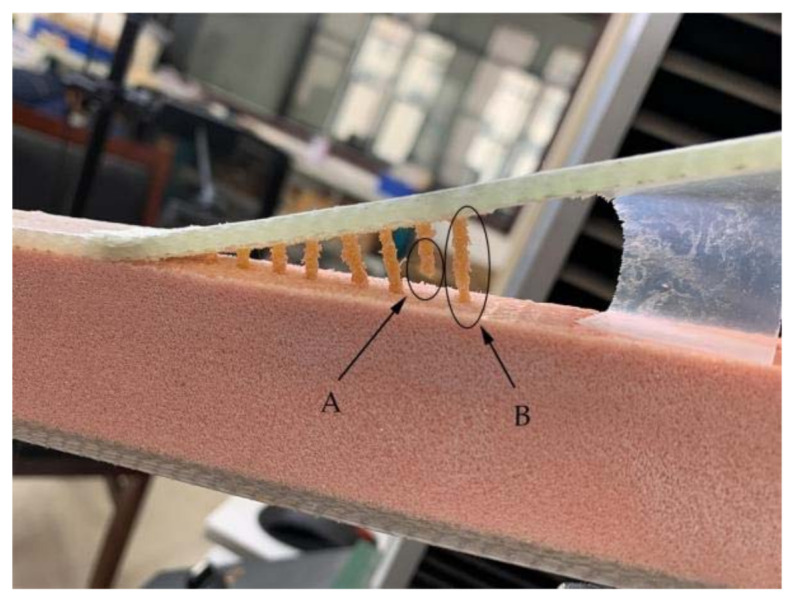
Fracture and pull-out of the resin column (A is the fracture of the resin column, and B is the pull-out of the resin column).

**Figure 10:**
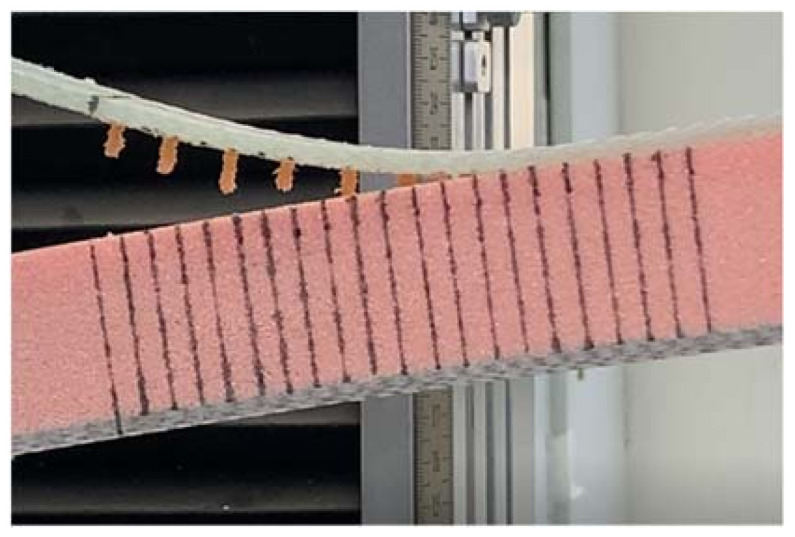
Testing of FH-10 specimen.

During the opening of the interface, the crack propagation in the stitched specimens needed to overcome not only the adhesion of the interface resin to the panel fibers and foam core, but also the pulling force of the resin column. Therefore, when the specimen interface was opened, the number of resin columns per unit area near the crack tip greatly influenced the ability of the material to resist further crack expansion. The resin columns here include both those in the pulling process and those waiting to be pulled out. For FH-6 and FH-8, the resin column only partially fractured during the loading process (Figure 9), where A is the fracture of the resin column, and B is the pull-out of the resin column, while for FH-10 the resin column fractured completely during the loading process (see Figure 10), which ended the unplugging process early. The reasons for the fracture of all resin columns in FH-10 can be explained as follows. The suture density and the local stiffness were low, whereas the local deformation was large. Therefore, when the first resin column started to be pulled out, the rear resin columns could not share the pulling force effectively due to the relatively long distance, resulting in the first resin column being pulled out at once.

The combined effects of potential energy accumulation and release frequency, potential energy cycle time span, potential energy accumulation in the initial linear phase, and column fracture in the unit area near the crack tip, eventually led to the fracture displacement in the following order: FH-10 > FH-6 > FH-8.

The peak load for FH-10 was lower than that for FH-6 and FH-8 and only slightly higher than that for FH-0 due to the premature end of the resin column pull-out process in FH-10. The peak load for FH-8 was higher than that for FH-6, this may be explained by the fact that FH-6 specimens absorbed more energy during the initial load linear increase stage (I), while the potential energy did not decrease to a large extent during crack propagation. Thus, the load–displacement curve for the FH-6 specimens showed a smaller load undulation during the crack propagation phase, compared with that for FH-8 specimens.

### 3.2. Crack Propagation Process

**Figure 11:**
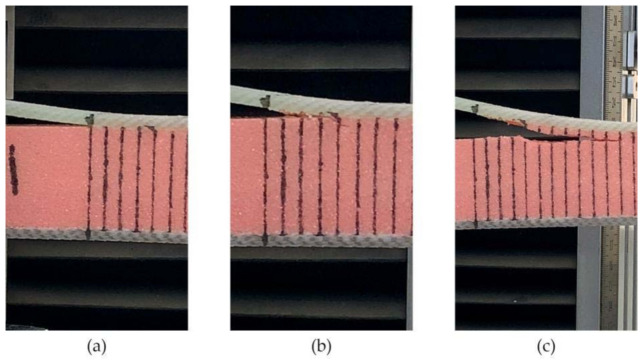
Crack propagation process in FH-0. (**a**) Crack initiation. (**b**) Pore-guided main crack bridging in the foam. (**c**) Smooth crack propagation in the foam.

**Figure 12:**
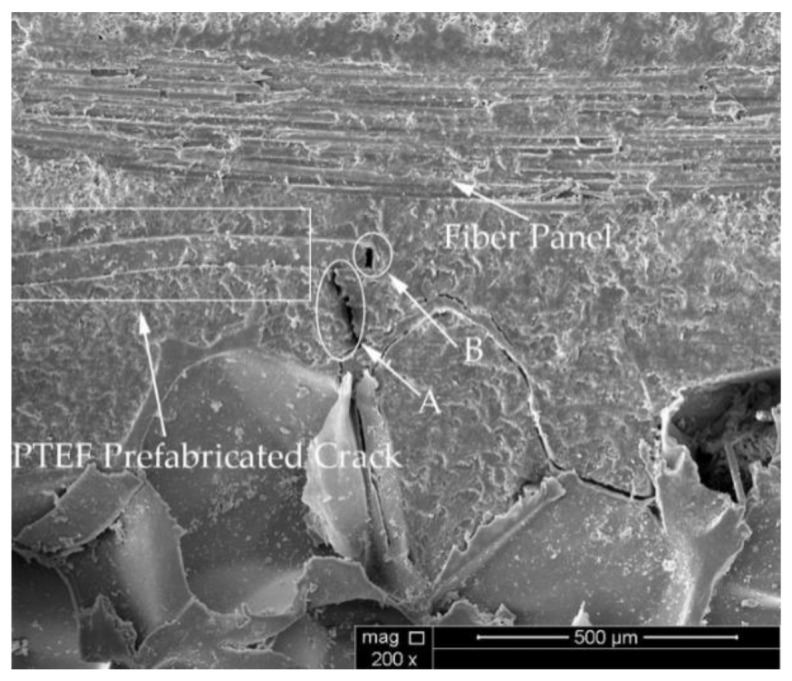
SEM images of FH-0 (A is the foam pore, and B is the crack initiation point).

**Figure 13:**
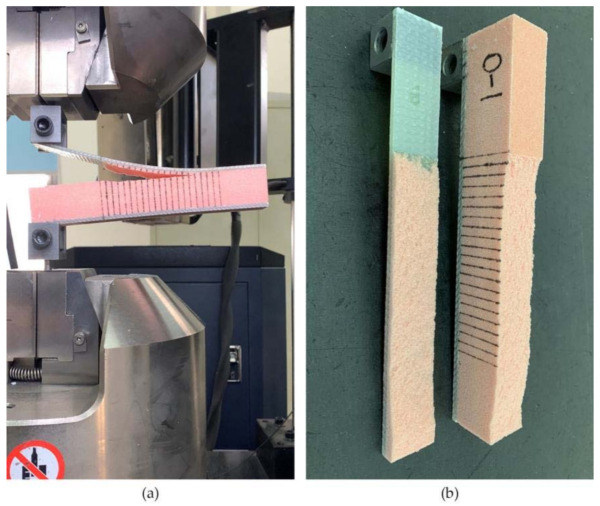
(**a**) Setup for FH-0-1 and (**b**) FH-0-1 specimen after testing.

During the loading process of the FH-0 specimens, the main crack started from the tip of the PTEF prefabricated crack (see Figure 11a). Since a low-density foam was used for the preparation of all specimens, the presence of many pores in the core material would affect the propagation direction of the main crack. After increasing the load, the main crack started to bridge with the foam pores (see Figure 11b), and the crack would expand smoothly at a distance of 2–3 mm from the interface (see Figure 11c). The bridging process between cracks and pores is demonstrated in Figure 12, where B is the crack initiation point and A is the foam pore. The crack started at B and was guided by pore A to expand to the foam side. The panel was still partially attached to the foam after the interface opening (see Figure 13), which is consistent with the fact that the material was made of low-density foam, where the weakest part would not be the interface but the foam. In fact, during the entire opening process of the specimens, the cracks mainly propagated in the foam.

**Figure 14:**
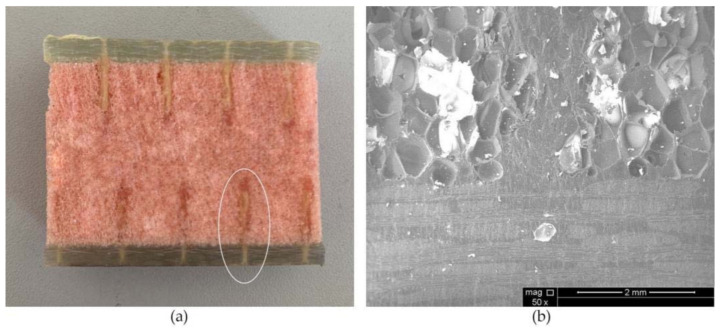
FH-8 specimen. (**a**) Suture profile and (**b**) SEM image of suture profile.

**Figure 15:**
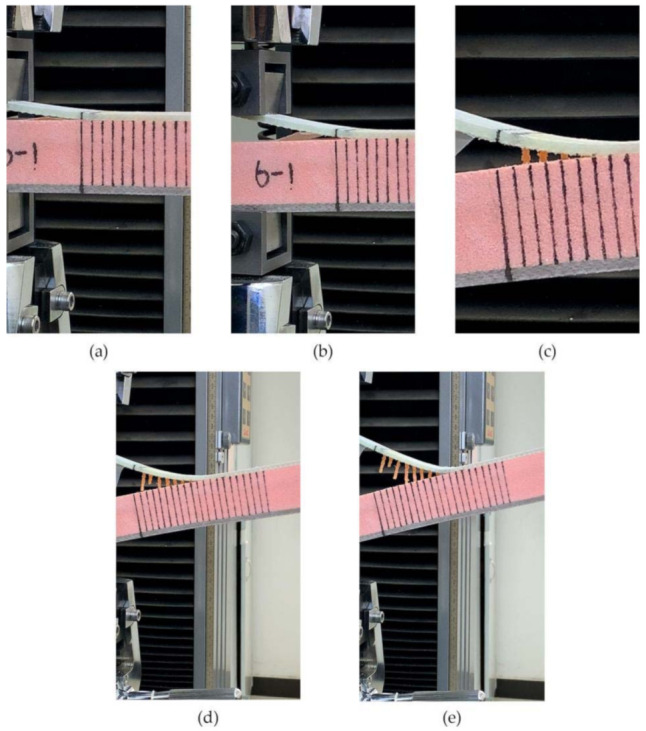
The crack propagation process in the sutured specimen. (**a**) Crack initiation. (**b**) Interface opening. (**c**) The leading edge resin column was pulled out or broken. (**d**) Interface opening. (**e**) The rear resin column was pulled out or broken.

The results for FH-8 were found to differ from FH-6. Due to the defects introduced by the stitching, a resin column/foam interface was formed in the foam (marked by the white circle in Figure 14a). This implies that the crack started and expanded at the resin column/foam interface, and subsequently the resin column was pulled out. A part of the resin column was fractured (see Figure 9), which resulted in a loud sound. In contrast to FH-0, the crack propagation in the FH-6, FH-8 and FH-10 specimens no longer needed to overcome the toughness of the foam core, but the adhesion of the resin to the panel fibers and the foam core, and the pulling force of the resin column. This result in cracks propagating along the interface in FH-6, FH-8 and FH-10 specimens, which suggested that the suture treatment had changed the crack propagation mechanism of the foam sandwich structures to a certain extent. The process of crack propagation in FH-0 followed the sequence of (i) Crack initiation (see Figure 11a) → (ii) Pore-guided main crack bridging in the foam (see Figure 11b) → (iii) Smooth crack propagation in the foam (see Figure 11c) → (iv) Finally the specimen was completely broken. The process of the crack propagation in the FH-6, FH-8 and FH-10 specimens was: (i) Crack initiation (see Figure 15a) → (ii) Interface opening (see Figure 15b) → (iii) The leading edge resin column was pulled out or broken (see Figure 15c) → (iv) Interface opening (see Figure 15d) → (v) The rear resin column was pulled out or broken (see Figure 15e) → (vi) Interface opening → (vii) The specimens were completely broken. It can be concluded that the introduction of the resin column caused changes in the crack propagation mechanism, which could be evidenced by comparing the sutured sample group and the un-sutured group. It was demonstrated that the crack inclined to propagate in the foam due to the guidance of the foam pores in FH-0, but to propagate along the interface between the panel and the foam in the sutured specimens.

### 3.3. Release Rate of Critical Strain Energy

The calculation of the strain energy release rate was carried out according to the modified beam theory method [24] as follows:

(1) $$G_{I}=\frac{3P\delta }{2ba}$$

where *P* is the load (N), *δ* is the loading point displacement (mm), *b* is the specimen width (mm) and *a* is the crack length (mm).

**Figure 16:**
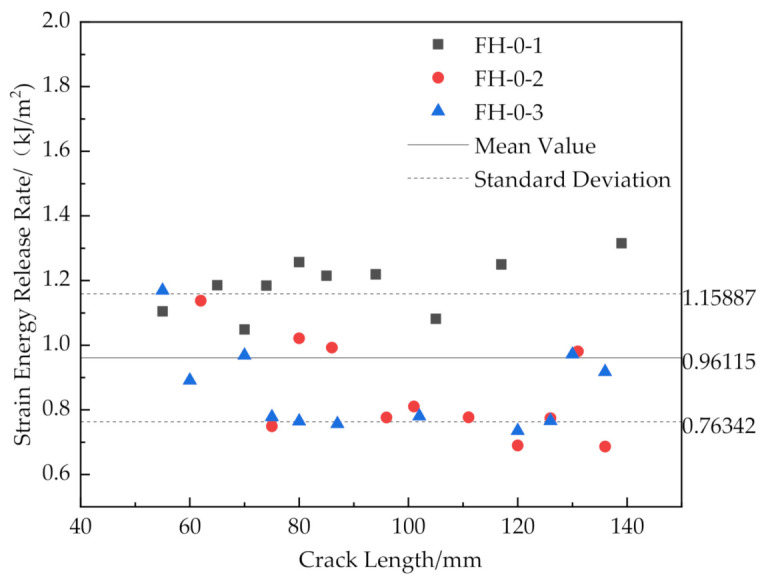
Release rate of critical strain energy of FH-0 with different crack lengths.

**Figure 17:**
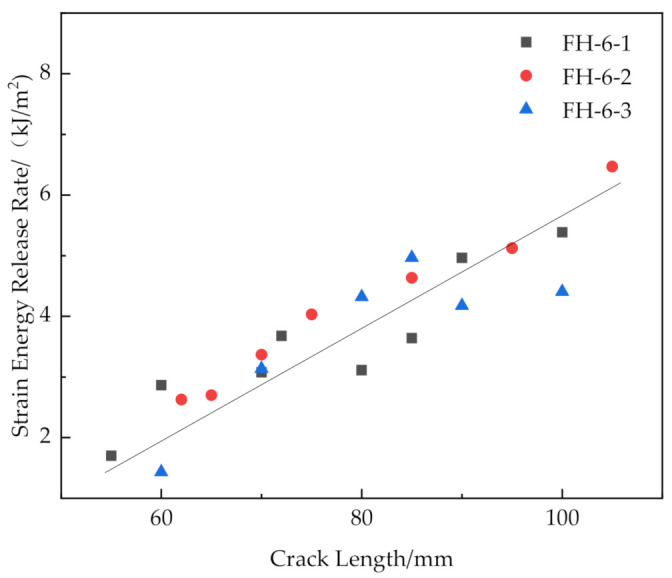
Release rate of critical strain energy of FH-6 with different crack lengths.

**Figure 18:**
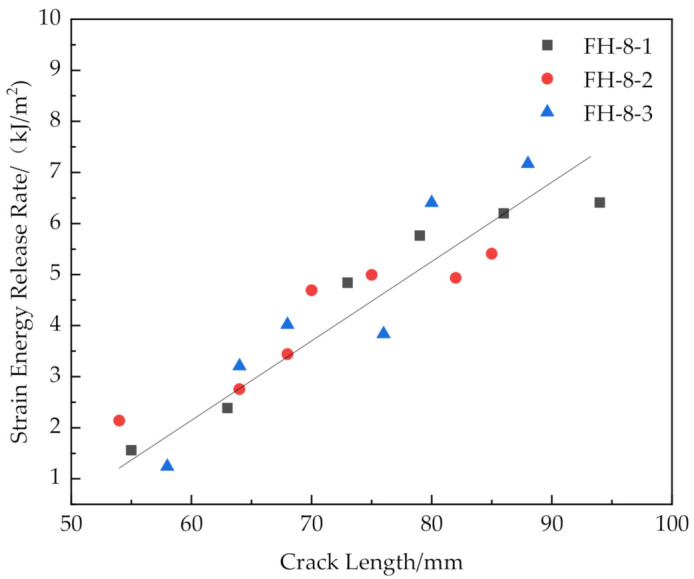
Release rate of critical strain energy of FH-8 with different crack lengths.

**Figure 19:**
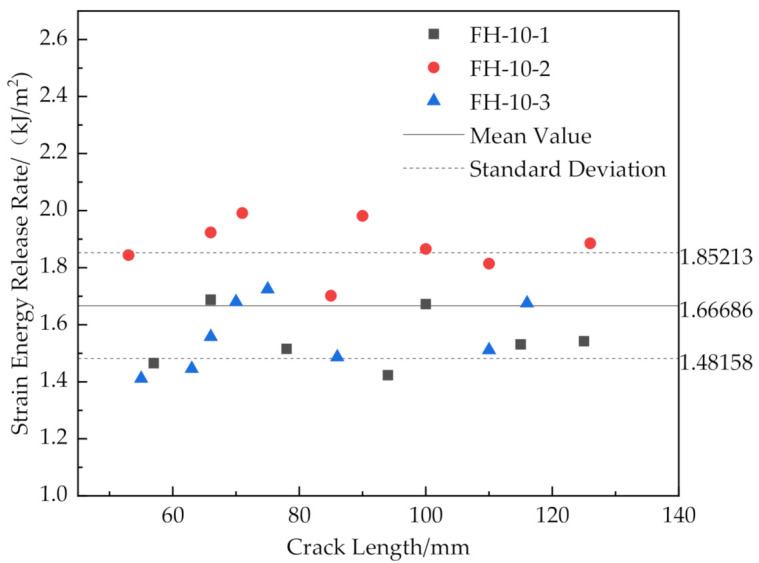
Release rate of critical strain energy of FH-10 with different crack lengths.

The results are shown in Figure 16, Figure 17, Figure 18 and Figure 19.

**Figure 20:**
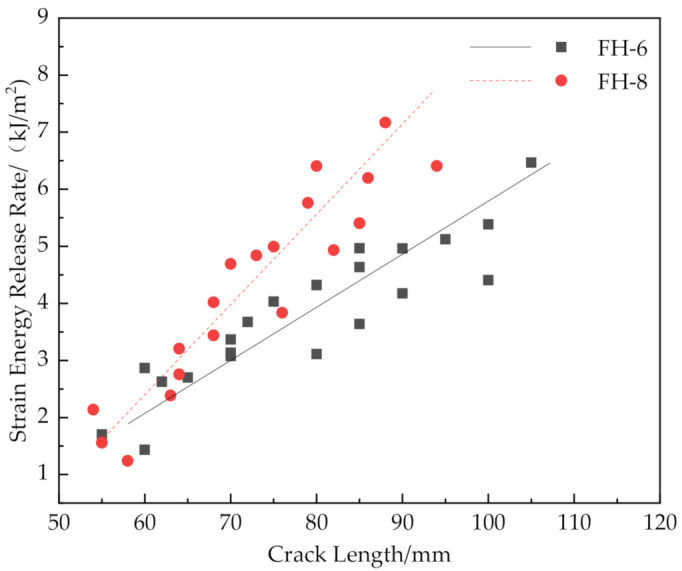
Comparison of release rate of the critical strain energy of FH-6 and FH-8 specimens with different crack lengths.

Figure 16 and Figure 19 show that the release rates of the critical strain energy-crack length distribution of the FH-0 and FH-10 groups were uniform with mean values of 0.961 and 1.667 kJ/m^2^, respectively. The stitching treatment with 10 mm spacing increased the average release rate of the critical strain energy by about 73.47%. This indicates that the larger stitching spacing only affected some of the material properties, such as the trend of load–displacement, the mean value of the release rate of the critical strain energy, whereas the trends in the curves of release rate of the critical strain energy vs. crack length did not change significantly and remained uniform. It can be seen from Figure 20 that the curves of release rates of the critical strain energy vs. crack length showed linear trends in FH-6 and FH-8.

The reasons for the difference in the strain energy release rate for each test group were the following: The release rate of the critical strain energy represents the energy required per unit area for the crack propagation of the material, which describes the resistance of the material to crack propagation and can, thus, be used to evaluate the fracture toughness of the material. During the opening of the interface, the crack propagation process of the sutured specimen needed to overcome the adhesion of the interface resin to the panel fibers and foam core and the pulling force of the resin column. Therefore, when the interface opened, the number of resin columns per unit area around the crack tip greatly affected the ability of the material to resist the further propagation of a crack. In the CSB tests, it was found that a few resin columns of FH-6 and FH-8 were fractured (see Figure 9), while all the resin columns of FH-10 were fully fractured (see Figure 10), which ended the pull-out process. After the fractured resin column was separated from the foam core, it no longer had any effect on the subsequent load–bearing process of the specimen, and the release rate of the critical strain energy was still distributed uniformly, similar to that of the unstitched specimen. Therefore, the distribution of the critical strain energy release rate of the specimens with suture spacing of 10 mm is still the same as that for FH-0 specimens, showing uniform distribution. Similarly, the effect of suture density on the process of crack propagation may explain the fact that the release rate of the critical strain energy in FH-8 was higher than that of FH-6. In summary, the stitching treatment with 8 mm spacing had the best suture toughening effect, the largest peak load and relatively high fracture displacement.

## 4. Conclusions

In this study, foam sandwich structures were sutured by a customized automatic reinforcement stitching equipment and the CSB test specimens were prepared. Mode-I CSB fracture tests were carried out. The effects of different stitch spacings on the load–displacement curves, crack propagation process and strain energy release rate were investigated. The following conclusions could be obtained:

1. The stitching treatment with different spacing increased the peak load and average fracture displacement of GFRP/foam sandwich composite, as revealed by CSB testing. The average peak loads of FH-6, FH-8 and FH-10 were 188.21, 239.81 and 139.47 N, respectively, which correspond to an 55.9%, 98.6% and 15.5% increase as compared to the average peak load of 120.75 N for FH-0. The average fracture displacements of FH-6, FH-8 and FH-10 were 61.34, 56.25 and 74.80 mm, respectively. The average fracture displacements increased by 28.8%, 18.1%, and 57.1%, respectively, compared with the average fracture displacement of 47.62 mm for FH-0.
2. In the CSB tests for FH-0, the crack expanded easily in the foam due to the guidance of the foam pores. The crack propagation mechanism in FH-0 specimens needed to overcome only the toughness of the foam core. However, in the sutured specimens, the crack propagated along the interface, where the forces to be overcome were the adhesion of the interface resin and the pulling force of the resin column.
3. The release rate of the critical strain energy for FH-10 was evenly distributed. The reason for this phenomenon was that the suture density was too low, and the adjacent resin columns could not share the pulling force effectively, so the resin column broke prematurely, and the fractured resin columns no longer affected the loading process after separation from the foam core. The release rate of critical strain energy for FH-6 and FH-8 were linearly distributed, and the absolute value and linear slope of the release rate of critical strain energy of FH-8 were higher, which was related to the high suture density of the FH-6 specimens.
4. FH-8 specimens had the best suture toughening effect and the largest peak load. Its fracture displacement was also improved to a certain extent, compared with that for FH-0. Overall, FH-8 had the best performance in all aspects. Concerning the actual production of sutured sandwich structures, it should be noted that the suture density does not linearly improve the interfacial fracture toughness of the materials, but there is an optimal suture spacing value remains to be tested.

## Data Availability

The data presented in this study are available on reasonable request from the corresponding author.
